# Gold nanoparticle-based colorimetric detection of ATP via a label-free aptamer

**DOI:** 10.3389/fbioe.2026.1871335

**Published:** 2026-08-14

**Authors:** Wenxiu Dai, Qingqin Hao

**Affiliations:** Department of Clinical Laboratory, Wuxi Blood Center, Wuxi, China

**Keywords:** aptamer, ATP, biosensor, colorimetry, gold nanoparticles

## Abstract

Adenosine triphosphate (ATP) is an important detection content in many biological reaction detection. At present, the detection methods of ATP mainly focused on electrophoresis, optical analysis, chromatography, and bioluminescence. However, these methods are not widely applied due to the high cost, complicated operation, time-consuming and low sensitivity. Here we developed a simple, fast and sensitive ATP biosensor with the specific aptamer as the recognition element and the unmodified gold nanoparticle (AuNPs) as the colorimetric probe. In the high salt environment, the aggregation of AuNPs causes the color change from red to blue. However, in the presence of aptamer, the free aptamers protect the AuNPs from aggregation and keeps the color of the solution red. Conversely, in the presence of ATP, aptamers are consumed to form aptamer-ATP complex, the AuNPs lose their protection and aggregate with the visual color change from red to blue. Our results show that ATP with a linear range of 5–320 μM can be detected. In conclusion, nanoparticles-based colorimetric method is feasible for ATP detection and has great potential in clinical screening.

## Introduction

Adenosine triphosphate (ATP), the basic energy exchange factor that connects anabolism and catabolism, is involved in the process of cell apoptosis and necrosis in living organisms (Bonora et al., 2012). Abnormal ATP level is closely associated with many diseases, such as hypoglycemia, cardiovascular and cancer (Cavaliere et al., 2002; Singareddy et al., 2022; Li et al., 2025). Hence, ATP is an important detection content in many biological reaction detection. At present, the detection methods of ATP mainly focused on electrophoresis, optical analysis, chromatography, and bioluminescence (Zinellu et al., 2010; Wang et al., 2013). However, these methods are not widely applied due to the high cost, complicated operation, time-consuming and low sensitivity. For example, bioluminescence method measures total organic material and ATP from all sources and could not distinguish between harmless organic debris and viable, pathogenic microorganisms. In addition, the residues of disinfectants, detergents, and sanitizers could alter luciferase enzyme reaction, resulting in false readings (Zhou et al., 2012; Arroyo et al., 2017). Therefore, increasing attention has been paid to finding fast, simple and sensitive ATP detection strategies.

After decades of development, aptamers have shown broad prospects in the application of biosensors and bioanalysis. Aptamer is a artificial single-stranded DNA (ssDNA)/RNA selected from random sequence nucleic acid libraries with SELEX (systematic evolution of ligands by exponential enrichment) techniques (Ellington and Szostak, 1990). Aptamer, also known as “chemical antibody,” works in a similar way to antibody target recognition (Toh et al., 2015). However, compared with antibodies, aptamers have the advantages of simple synthesis and modification, easy labeling, good stability and wide target range (Kim et al., 2016; Nimjee et al., 2005). In addition, aptamer has high affinity and high specificity for small molecules, proteins and even viruses and cells (Rozenblum et al., 2016), Therefore, aptamer as a small molecular sensor has shown broad application prospects in the field of biosensors and bioanalysis.

Colorimetric assay is a widely used detection assay (Li et al., 2012; Charych et al., 1993). Due to its unique physical and chemical properties, including high extinction coefficient in visible light region, surface plasmon resonance (SPR) absorption, simple preparation and easy functionalization, the colorimetric assay based on gold nanoparticles has attracted much attention (Cao et al., 2017; Zhao et al., 2007; Riham and Digambara, 2018). Gold nanoparticle (AuNP) colorimetric biosensor is highly advantageous due to rapid, low-cost, on-site detection with naked-eye visualization, eliminating the need for complex laboratory instrumentation. The core sensing mechanism relies on a direct, logical relationship between salt-induced instability and ssDNA protection (Lian et al., 2004). Of note, the stability of the gold nanoparticles was easily affected, resulting in the aggregation of AuNPs changed to blue, but the AuNPs could be stabilized by ssDNA, so that the AuNPs returned to red.

Here we report a colorimetric method for ATP detection using label-free aptamer as the probe and unmodified AuNPs as the indicators. ATP is detected by monitoring the color change of AuNPs and spectrophotometry. The aptamer was screened out using capillary electrophoresis (CE) SELEX Technology which can be adsorbed on the surface of the AuNPs in the salt solution to prevent its aggregation (Li et al., 2009). However, in the presence of ATP, the aptamer dissociates the AuNPs and binds to the ATP, which causes the color to change from red to blue. By optimizing several key variables, a colorimetric method for sensitive and rapid determination of ATP was established.

## Experimental

### Reagents and chemicals

ATP was purchased from Takara Biotechnology (Dalian, China). Guanosine triphosphate (GTP), cytidine triphosphate (CTP), uridine triphosphate (UTP), adenosine diphosphate (ADP) and adenosine monophosphate (AMP) were purchased from Sangon Biotech (Shanghai, China). HAuCl_4_.4H_2_O was purchased from West Asia Chemical Industry Co., Ltd (Shandong, China). Sodium citrate and other chemical reagents were supplied by Tianjin bodi chemical Co., Ltd (Tianjin, China). All oligonucleotides were synthesized and purified by Sangon Biotech. The aptamer sequence was as follows. ATP aptamer: 5′-C CTG GGG GAG TAT TGC GGA GGA AGG T-3’.

### Apparatus

The size of AuNPs was measured by transmission electron microscope (TEM, Hatichi, Japan). Absorbancy data were collected with a spectrophotometer (Thermo Multiskan Go, USA).

### Preparation of AuNPs

**FIGURE 1:**
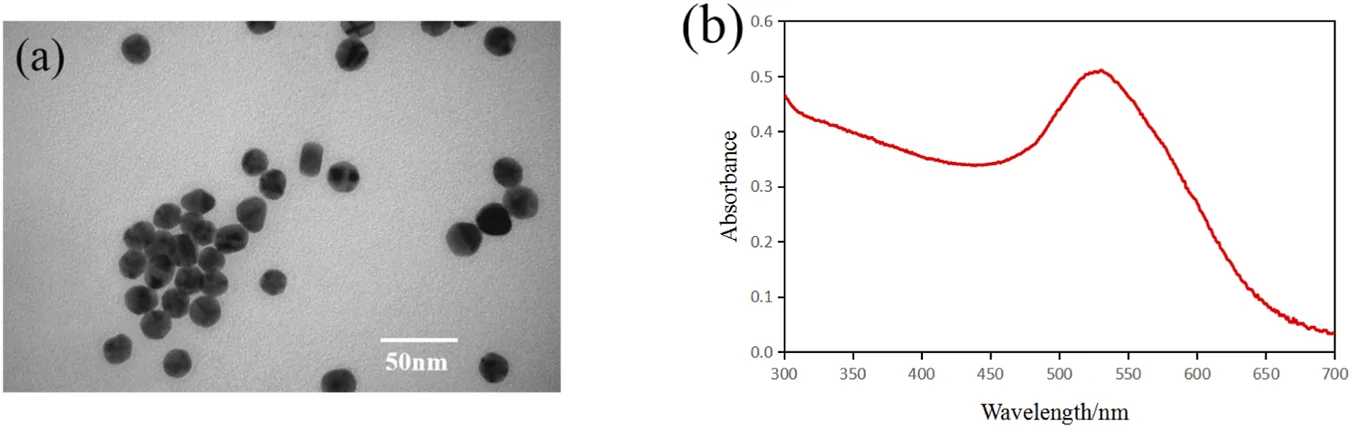
The characterization of prepared AuNPs. **(a)** TEM image of prepared AuNPs. **(b)** The absorption spectra of the synthesized AuNPs.

AuNPs were synthesized by citrate reduction of HAuCl_4_. Briefly, 50 mL HAuCl_4_ (1 mM) solution was heated to boiling in a 100 mL round-bottomed flask. Then a total of 5 mL of 38.8 mM sodium citrate was added rapidly to the solution with continuous heating for 10 minutes. After cooling, the solution was filtered through a 0.22 μm filter and stored in dark bottles at 4 °C. The characterization of prepared AuNPs was shown in Figure 1. The average size measured by TEM is about 13 nm (Figure 1a). The absorption spectra of the synthesized AuNPs was measured, and the concentration of AuNPs was quantitatively determined at 520 nm according to the molar extinction coefficient of 2.5 × 10^8^ L mol^-1^ cm^-1^ (Figure 1b).

### Colorimetric detection of ATP

In order to establish AuNPs based colorimetric sensor for detecting ATP, the experimental system was described as follow: In brief, lyophilized ATP aptamer was dissolved in 10 mM PBS buffer. Then 20 μL of PBS buffer containing ATP aptamer or containing aptamer and different concentrations of ATP (final 5, 10, 20, 40, 80, 160, and 320 μM) were added to 80 μL of AuNPs solution and incubated at room temperature for 30 min. Subsequently, 5 μL of 60 mM NaCl was mixed with the solution to provide a final volume of 105 µL. The spectra values were collected at the range of 300–700 nm.

### Specificity assay

ATP analogues including GTP, CTP, UTP, ADP and AMP were quantitatively analyzed to evaluate the specificity of the aptasensor. The detection procedure was similar to that of ATP. In short, the target molecules in the appeal system were replaced with GTP, CTP, UTP, ADP and AMP with the remaining solution concentrations unchanged.

## Results and discussion

### Principles of the colorimetric method for ATP

**SCHEME 1:**
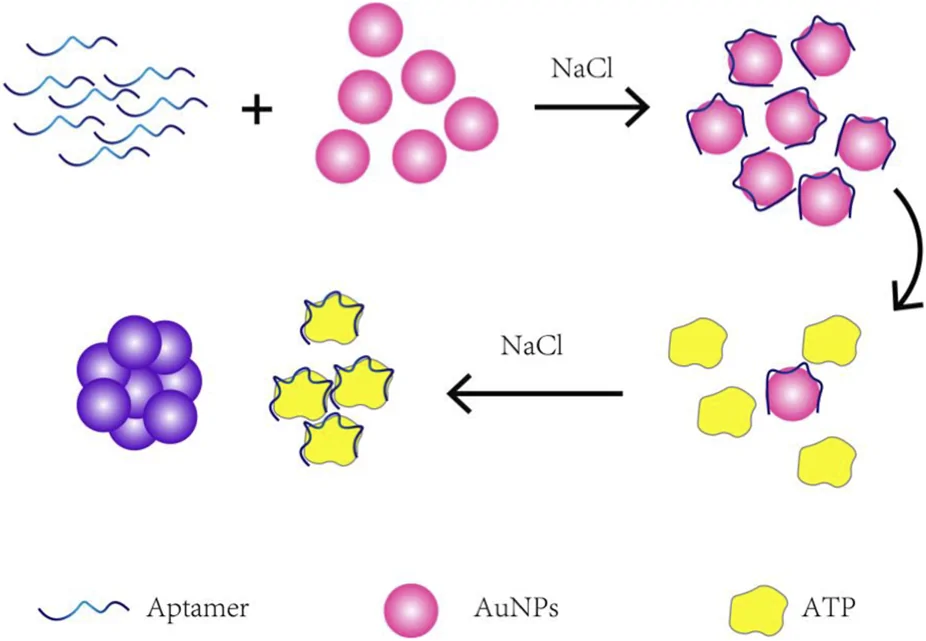
Schematic diagram of ATP detection by colorimetric method based on specific aptamer and AuNPs.

In our study, the AuNPs probes were synthesized by reducing HAuCl_4_. The AuNPs are uniformly dispersed through the van der Waals force between the nanoparticles. It is common knowledge that the surface of AuNPs could be absorbed by dissociative aptamers to hold back the aggregation between nanoparticles in NaCl environmen. In the presence of targets, by virtue of the interaction between aptamers and targets, the aptamers desorb from AuNPs and binds to ATP (Mondal et al., 2018). The aggregation of AuNPs in salt solution resulting in the visible color change from red to blue (Scheme 1). The sample solution was measured with a spectrophotometer, and the ATP absorbance was measured in the wavelength range of 300 nm and 700 nm.

**FIGURE 2:**
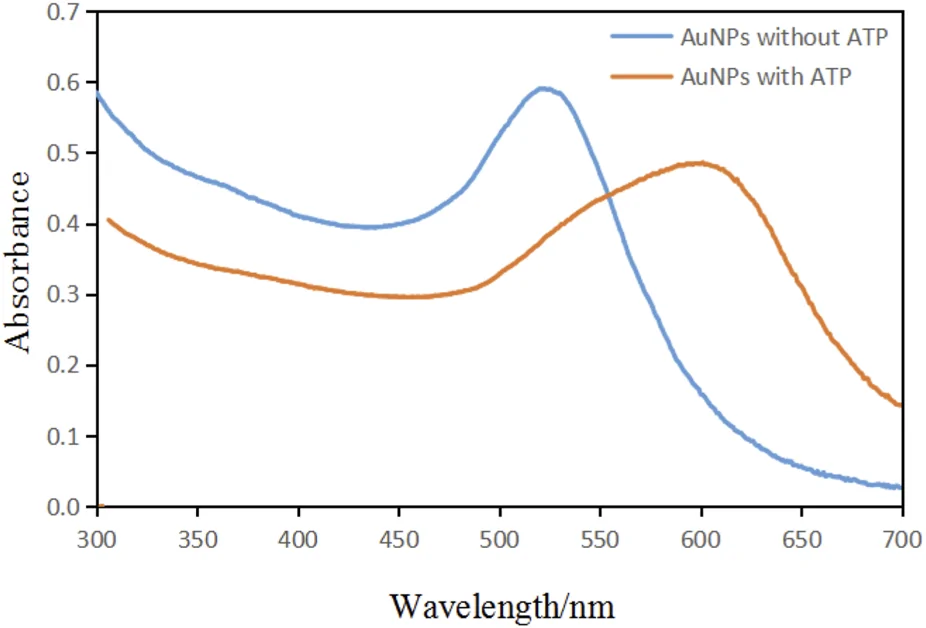
Feasibility of this assay.

**FIGURE 3:**
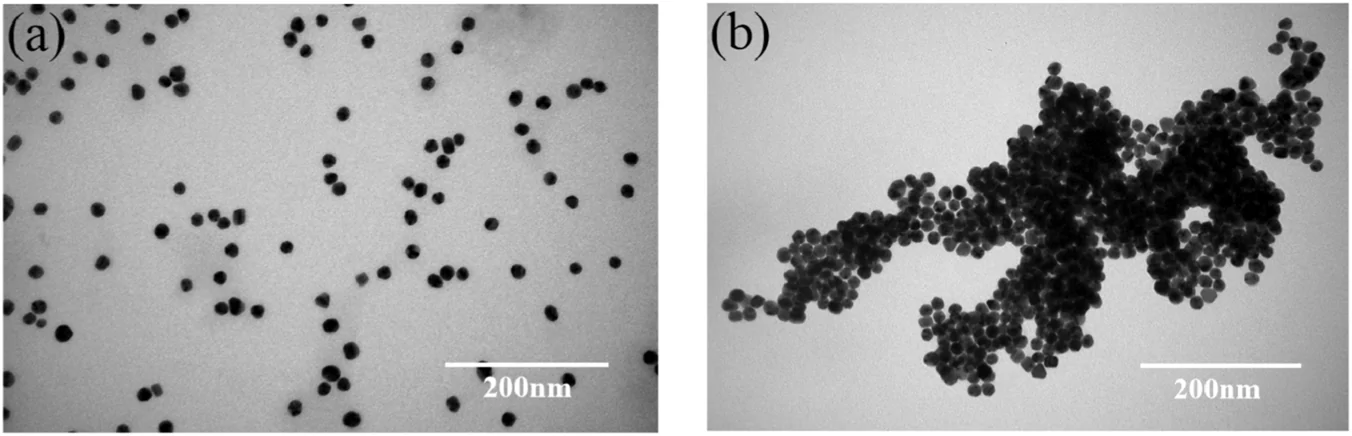
TEM images of the AuNPs solution mixed with ATP’s aptamer in the absence **(a)** or presence **(b)** of 50 μM ATP after the addition of 60 mM NaCl.

It can be seen from Figure 2 that in the salt solution, aptamer and AuNPs mixed solution was detected by the spectrophotometer when the ATP was absent or present. In addition, the spectrophotometer detects peak shift in the presence of ATP. A new peak was detected at 592 nm, which was consistent with the image results of TEM as shown in Figure 3, proving the feasibility of this experiment.

### Optimization of assay condition

**FIGURE 4:**
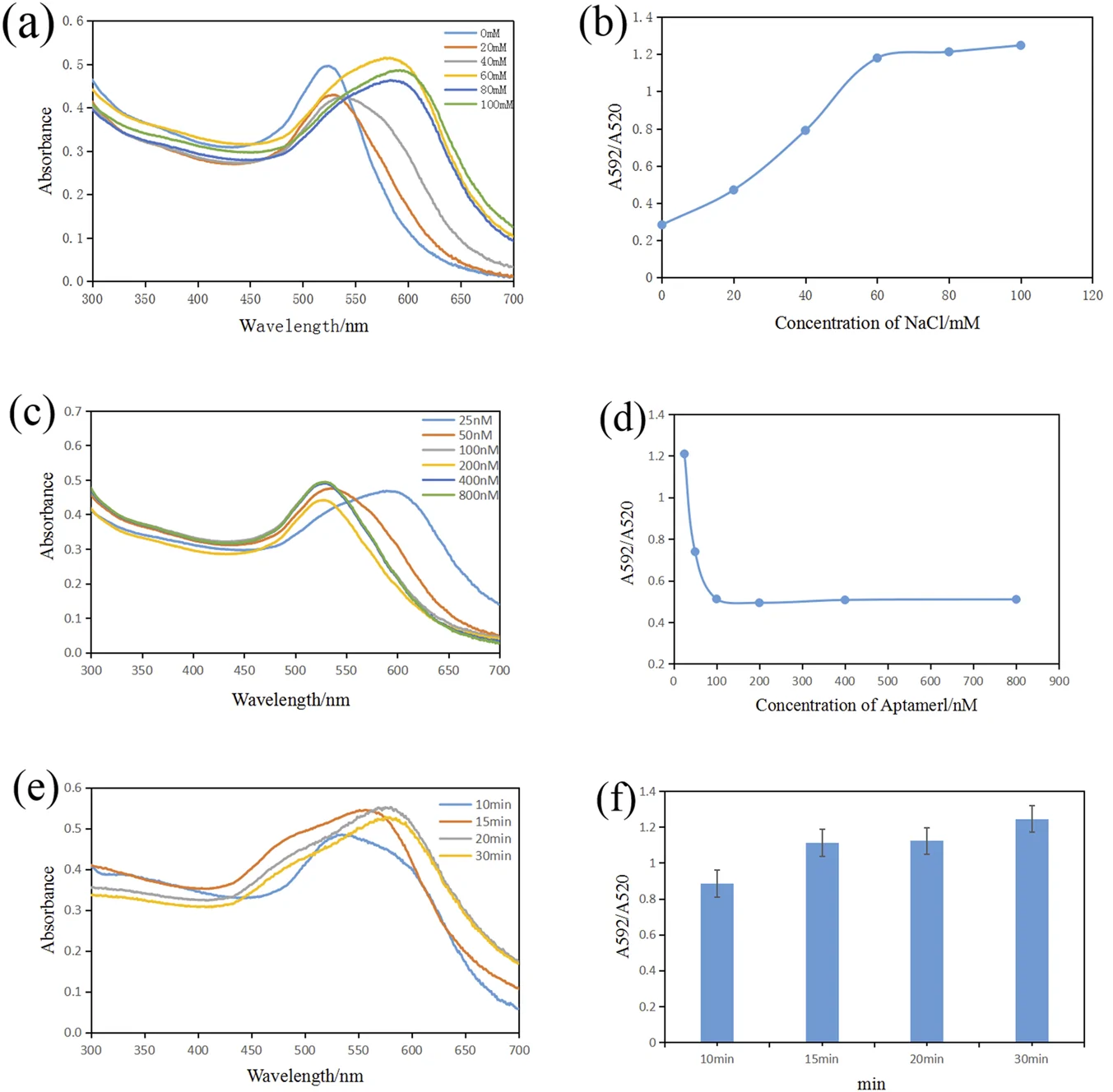
Optimization of key variables of the colorimetric conditions. **(a)** Absorption spectra of reaction at various NaCl concentration. **(b)** Absorbance ratio (A592/520) of NaCl concentration (0, 20, 40, 60, 80, 100 mM). **(c)** Absorption spectra for reaction with different concentrations of aptamer under the optimal NaCl solution. **(d)** Absorbance ratio (A592/520) of concentration of aptamer (25, 50, 100, 200, 400, 800 nM). **(e)** Absorption spectra of different time of reactions between aptamer and ATP under the optimal NaCl and aptamer conditions. **(f)** Absorbance ratio (A592/520) of reaction time (10, 15, 20, 30 min) between aptamer (100 nM) and ATP (50 μM). All data were from 3 independent experiments.

In order to obtain rigorous results, we need to optimize the key variables. Salt concentration is an important factor affecting AuNPs aggregation, low concentrations of salt do not cause color change, while high concentrations of salt can cause rapid aggregation of AuNPs accompanying with the color change from red to blue, so the optimal salt concentration needs to be determined first. Herin, we mixed 5 μL different concentrations (0, 20, 40, 60, 80, 100 mM) of NaCl and 80 μL AuNPs for 5 min. The absorbance values from 300 to 700 nm were revealed in Figure 4a. With the increase of salt concentration, the absorbance of AuNPs at 520 nm decreased and the peak values offset to 592 nm. When the concentration of NaCl reached to 60 mM, the absorbance ratio (A592/A520) reaches the maximum (Figure 4b). Thus, 60 mM NaCl was suitable for aggregating all AuNPs and induces obvious color change under the presence and absence of ATP.

Secondly, the aptamer concentration is one of the important variables. In the salt solution, the low concentration of the aptamer cannot prevent the concentration of AuNPs. On the contrary, the large background signal generated by excessive aptamers may reduce the sensitivity of the probe, which is not conducive to the detection of target molecules. Therefore, the absorbance was detected in the range of 300–700 nm by adding aptamers of different concentrations (25, 50, 100, 200, 400, 800 nM) in the mixed solution. As shown in Figure 4c, the absorbance ratio (A592/A520) decreases with the increase of the aptamer concentration, and tends to be stable when the aptamer concentration reaches 100 nM (Figure 4d). Therefore, 100 nM is the optimal concentration to detect ATP.

Finally, the reaction time of aptamer and ATP was optimized. 50 μM ATP was added to the mixed solution, and incubated at different times to measure the absorbance in the range of 300–700 nm. As shown in Figure 4e, the absorbance also showed differences with different incubation times. When the incubation time is short, and the difference in absorbance was probably not obvious because the aptamer had not been tightly bound to ATP. Similarly, the long incubation time and the tight binding of the aptamer to ATP make it difficult for AuNPs to obtain the protection of the adaptor through competition. Therefore, 15 min is the most suitable incubation time for later detections (Figure 4f).

### Assay linearity and sensitivity

**FIGURE 5:**
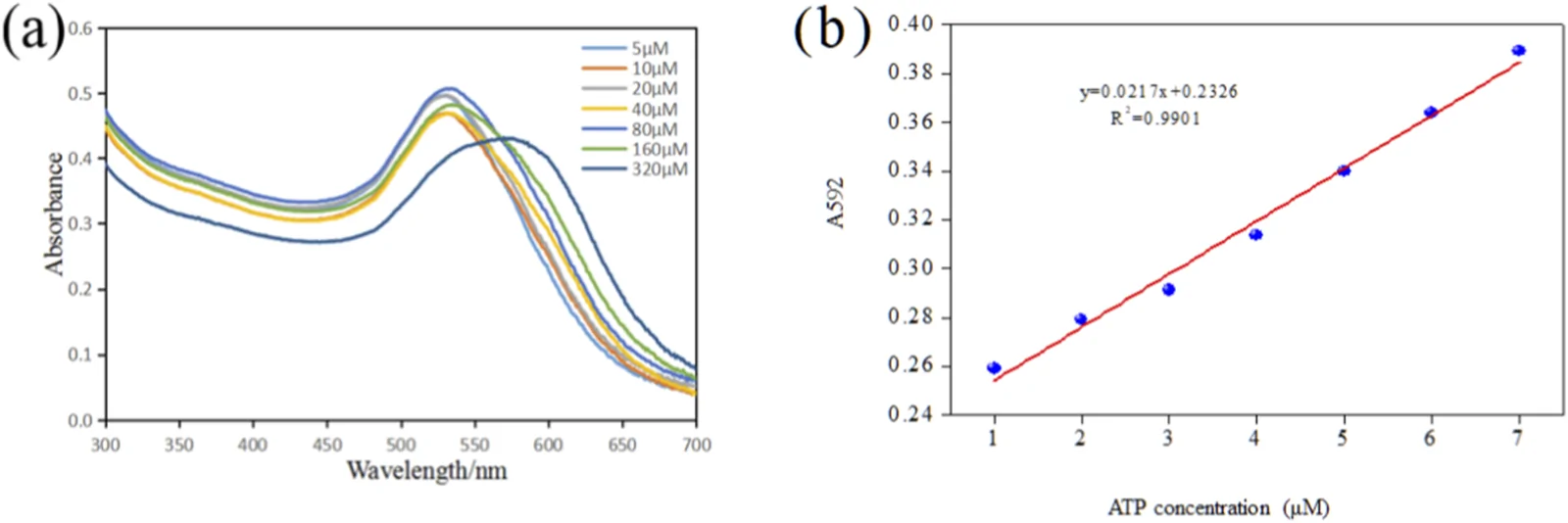
A colorimetric assay of ATP under the optimized experiment conditions. **(a)** Absorption spectra of the AuNPs when colorimetric probes was used for various concentrations of ATP (from up to down 5, 10, 20, 40, 80, 160, and 320 μM). **(b)** A592 values were directly extracted from the UV-vis spectra presented in Figure 5a, and a linear correlation was shown in presence of various concentrations of ATP. All data were from 3 independent experiments.

To summarize, the optimized experimental conditions are as follows: 1. a 60 mM concentration of NaCl; 2. a 100 nM concentration of aptamer; 3. a 15 min incubation time of aptamer with ATP. Under the experimental conditions of optimized conditions, the effects of different concentrations of ATP on the absorbance of AuNPs were investigated. As shown in Figure 5a, as ATP concentration increases, the absorbance peak decreases at 520 nm and shifts to 592 nm, causing the color change from red to blue. As shown in Figure 5b, A592 is linearly correlated with ATP concentrations in the ranges of 5, 10, 20, 40, 80, 160, and 320 μM, indicating that we have established a method for detecting ATP based on unmodified AuNPs and specific adaptors.

### Assay selectivity

**FIGURE 6:**
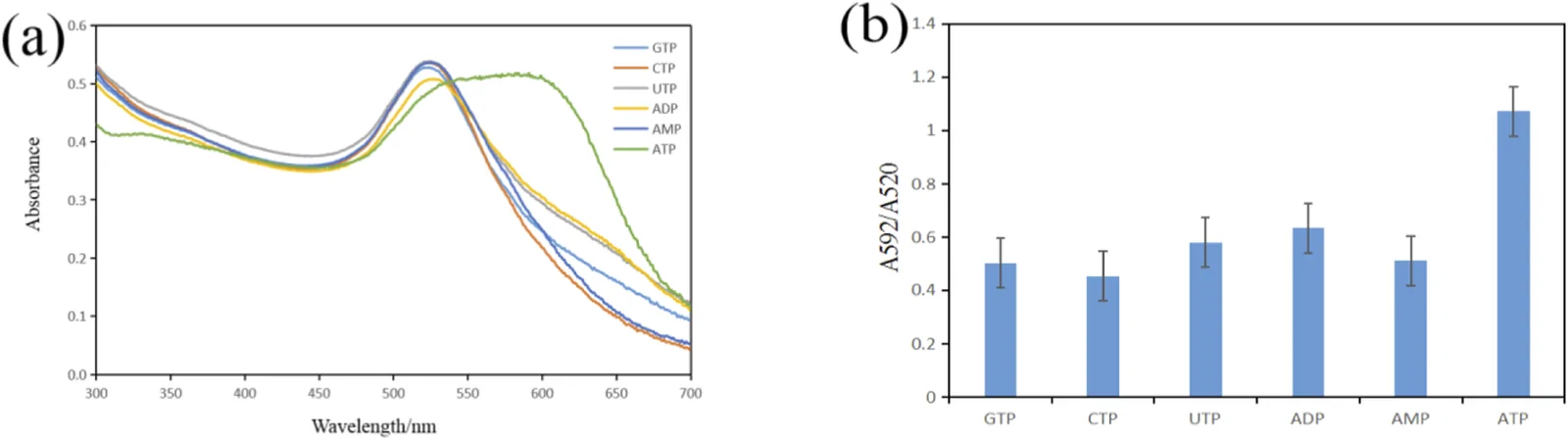
Selectivity of the aptamer-based assay for ATP detection. **(a)** Absorption spectra of AuNPs/aptamer mixed solutions in the presence of ATP and other ATP analogues. **(b)** Absorbance ratio (A592/520) of AuNPs/aptamer mixed solutions in the presence of ATP and other ATP analogues. All data were from 3 independent experiemnts.

The specificity of the colorimetric biosensor was evaluated by detection with other ATP analogues, including GTP, CTP, UTP, ADP and AMP. In the aptamer-AuNPs-NaCl system, only the addition of ATP can observe the reactivity and aggregation of AuNPs (Figure 6). The results revealed that the colorimetric biosensor showed good specificity in detecting ATP.

## Conclusion

In summary, a colorimetric method based on unmodified AuNPs and specific aptamer was developed to detect ATP rapidly and sensitively. This method is simple and time - saving, and the results are visible to the naked eye. By optimizing the salt concentration, the amount of aptamer and the incubation time of aptamer with ATP, ATP with a linear range of 5–320 μM can be detected. This method does not require expensive instruments and can detect results within 30 min, which has potential application in clinical practice.

## Data Availability

The data and material generated or analyzed in this study are available upon reasonable request, and could be provided by Qingqin Hao (hu78h87h@gmail.com or gap369@126.com).
